# Evaluation of the Effect of Using Different Types of Clinker Grinding Aids on Grinding Performance by Numerical Analysis

**DOI:** 10.3390/ma18122712

**Published:** 2025-06-09

**Authors:** Yahya Kaya, Veysel Kobya, Murat Eser, Naz Mardani, Metin Bilgin, Ali Mardani

**Affiliations:** 1 Department of Civil Engineering, Bursa Uludag University, Bursa 16059, Turkey; yahyakaya00@gmail.com (Y.K.); v.kobya@gmail.com (V.K.); 2 Department of Computer Engineering, Canakkale Onsekiz Mart University, Canakkale 17010, Turkey; meser@comu.edu.tr; 3 Department of Mathematics Education, Bursa Uludag University, Bursa 16059, Turkey; nazmardani@uludag.edu.tr; 4 Department of Computer Engineering, Bursa Uludag University, Bursa 16059, Turkey; metinbilgin@uludag.edu.tr

**Keywords:** grinding efficiency, grinding aids, Blaine fineness, artificial neural networks, TabNet, XGBoost, Random Forest, machine learning, regression analysis

## Abstract

To develop more environmentally friendly and sustainable cementitious systems, the use of grinding aids (GAs) during the clinker grinding process has increasingly gained attention. Although the mechanisms of the action of grinding aids (GAs) are known, the selection of an effective grinding aid (GA) can be difficult due to the complexity of appropriate selection criteria. For this reason, it is important to model the effect of GA properties on grinding performance. In this study, seven different types of GAs were used in four different dosages, and time-dependent grinding was performed. The Blaine fineness values of cements were compared after each grinding process. In addition, the modeling of these parameters using machine learning and ensemble learning methods was discussed. The Synthetic Minority Over-sampling Technique (Smote) was used to generate artificial data and increase the number of data for the grinding efficiency experiment. The data were modeled using methods such as Artificial Neural Networks (ANNs), Attentive Interpretable Tabular Learning (TabNet), Random Forests (RFs), and the XGBoost Regressor (XGBoost), and the ranking of the parameters affecting the Blaine properties was determined using the XGBoost method. The XGBoost method achieved the best results in the MAE, RMSE, and LogCosh metrics with values of 21.0384, 33.7379, and 15.4846, respectively, in the experimental modeling studies with augmented data. This study contributes to a better understanding of the relationship between GA selection and milling process performance.

## 1. Introduction

The growing demand for cement contributes significantly to environmental issues, primarily due to CO_2_ emissions generated during its production. Various strategies have been adopted to mitigate the adverse environmental impacts associated with cement use. One such approach involves enhancing the compatibility between cement and water-reducing admixtures to enable more sustainable and eco-friendly production processes [1,2,3,4,5,6]. Another strategy focuses on the partial or complete replacement of cement with alternative binders [7,8,9,10,11]. A third method is the incorporation of grinding aids (GAs) during the cement grinding process, which can enhance both grinding efficiency and the performance characteristics of cement [12,13,14].

GAs are used to save energy and reduce greenhouse gas emissions during clinker grinding [15,16]. GAs are adsorbed on clinker particle surfaces due to their high-polarity functional groups (-OH, -NH_2_, -COOR, -SO_3_, etc.) [17,18]. An adsorbed GA neutralizes the electrical charges on the surface and prevents clinker cracks from closing and particles from coalescing and sticking to the mill surface and/or balls [17,19]. This saves energy during the grinding stage. In clinker grinding, amine and glycol-based GAs are generally used to save energy [20,21,22,23]. Along with the development of this technology, various modification studies have been carried out to improve the performance of GAs. For instance, some studies found that the hydroxyl groups of GAs are enhanced by various modification processes to improve their performance [24,25].

Although the mechanisms of action of GAs are known, the selection of these additives is still based on hypothetical information. In particular, due to the complexity of the appropriate selection criteria, the selection of a performance-efficient grinding aid is extremely difficult. Many parameters such as molecular weight, number of hydrogen bonds, pH, number of functional groups, etc., can affect grinding performance. For this reason, modeling the effect of GA properties on grinding performance becomes important in terms of both time and cost. Modeling these parameters can be achieved using two different learning methods, machine learning and ensemble learning. Machine learning is a field of study that focuses on developing algorithms and models that allow computers to learn and make predictions or decisions without being explicitly programmed. It is also used in natural language processing, computer vision, regression problems, fraud detection, recommender systems, and many other areas [26,27,28,29,30,31]. It performs statistical computations on data to detect, learn, and predict specific patterns in a dataset. Ensemble learning is an approach to machine learning that makes predictions based on the decisions of multiple learning algorithms or learning methods that come together to solve a problem. The ensemble methods are trained separately to solve the problem. In the decision phase, a final decision is made based on the outputs produced by the methods [32,33]. The main purpose of its use is to try to increase the prediction success of the method. Some modeling studies on GAs and the grinding process are given in Table 1.

This study addresses a critical challenge in modeling experimental data for grinding efficiency: the limited size of the original dataset. To overcome this, the Synthetic Minority Over-sampling Technique (SMOTE) was employed to generate high-quality artificial data, effectively enriching the dataset and enhancing model robustness. Unlike simple data duplication, SMOTE innovatively creates new synthetic samples by interpolating between existing minority class data points using K-Nearest Neighbors, thus preserving the underlying data distribution and improving learning outcomes [39]. Leveraging this augmented dataset, grinding efficiency was modeled using a combination of advanced learning techniques, including Artificial Neural Networks (ANNs) as a traditional machine learning method, the transformer-based Attentive Interpretable Tabular Learning (TabNet) for deep learning, and ensemble methods such as Random Forest (RF) and the XGBoost Regressor. This multi-method approach not only improves prediction accuracy but also provides comprehensive insights into the factors controlling Blaine fineness, with the Random Forest model specifically employed to rank the dominance of input parameters influencing grinding performance. The integration of SMOTE with diverse state-of-the-art modeling techniques in this work represents a novel and systematic strategy to optimize data-driven predictions in grinding aid research, contributing valuable methodological advancements to the field.

## 2. Materials and Methods

### 2.1. Materials

**Figure 1:**
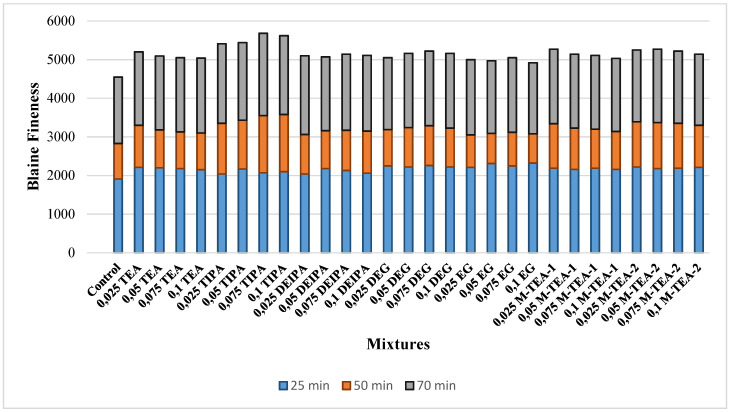
Blaine fineness values of cement prepared with different grinding times, GA type, and dosage.

Cements containing different types and proportions of GA were produced as part of this study. For this purpose, 96% clinker and 4% gypsum were used. A total of 85 cements with a GA were prepared following the EN 197-1 standard [40] by using different GAs during grinding processes carried out at three different grinding times in a laboratory-type ball mill. Accordingly, in addition to the commonly used commercial GAs based on triethanolamine (TEA), triisopropanolamine (TIPA), diethanolisopraponalamine (DEIPA), diethylene glycol (DEG), and ethylene glycol (EG), a total of 7 different GAs, including two different modified TEAs obtained by the esterification reaction of a TEA-type GA, were added to the mixture during the cement grinding stage. All the GAs were used in proportions of 0.025%, 0.05%, 0.075%, and 0.1% of the total clinker and gypsum mass. The properties of the GAs used are shown in Table 2, and the Blaine finenesses of each cement after grinding at three different grinding times are shown in Figure 1. The cements were named according to the type and dosage of the GA used. For example, the cement prepared with 0.025% TEA was named 0.025 TEA.

### 2.2. Method

**Figure 2:**
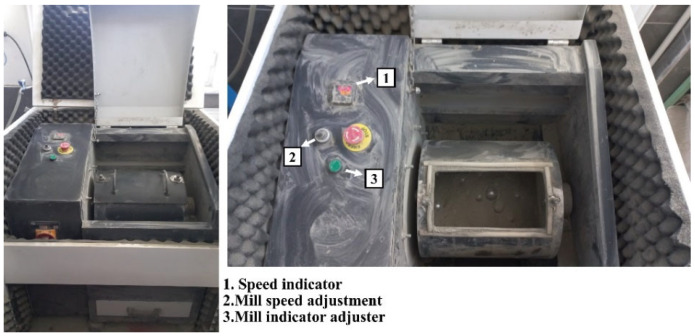
Laboratory type mill where the grinding process is carried out.

**Figure 3:**
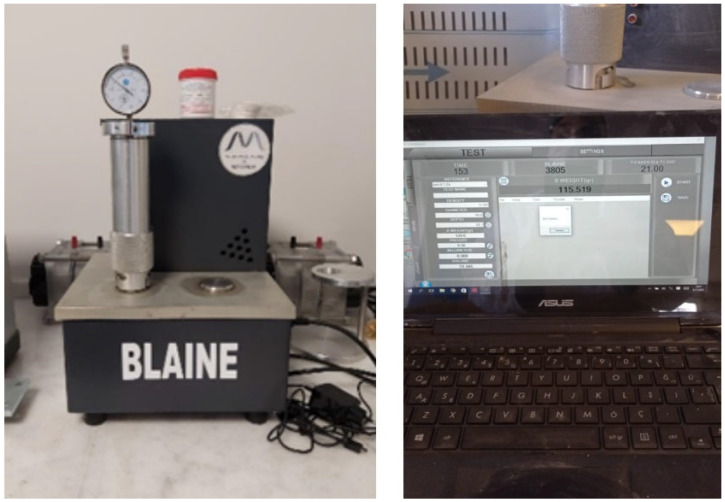
Automatic Blaine device.

Cement production was carried out using a laboratory-type ball mill with 1.5 kW motor power, 35 rpm rotation speed, 19 cm width, 48 cm diameter, and 5 kg capacity (Figure 2). Clinker and gypsum were prepared according to the Bond Standard before grinding. Ball distribution was also selected according to the Bond Standard. In addition, the Blaine fineness of the ground cements was measured using an automatic Blaine apparatus, depicted in Figure 3.

#### Methodology of Modeling

Information on the modeling of Blaine data obtained as a result of experimental studies using 8 different methods in a computer environment is presented. The 84 data obtained from the experimental studies were used in the modeling studies. To create more efficient models, the input data were first normalized between 0.1 and 0.9. The corresponding formula is shown in Equation (1). Since machine learning algorithms are sensitive to the differences between the scales of the input data, learning with unscaled data usually results in poor performance. In multivariate regression problems, normalization is used to improve performance so that variables in different scale ranges contribute equally to the analysis. For example, if normalization is not applied to a feature between 0 and 1000 and a feature between 0 and 10 in the dataset, the distribution of weights for the model will be disproportionate. For this reason, the goal is to increase the success rate by applying the normalization process to experimental data.

(1) $$\begin{array}{c} O_{i}=0.8\frac{\left(O-O_{min}\right)}{\left(O_{max}-O_{min}\right)}+1+0.1\; \end{array}$$

Before computer modeling of the Blaine fineness values of the cements obtained after grinding, artificial data were generated by the Smote method using the available data. After the data augmentation process, the number of data for Blaine experiments was increased from 84 to 1500. For the determination of the hyperparameters of the XGBoost and RF models, a K-fold cross-validation technique was used to ensure that all data were available in both the training and test sets. K-fold cross-validation is a widely used technique for estimating the performance of a machine learning algorithm on a given dataset. It involves dividing the dataset into K subsets, using K-1 subsets for training, and the remaining subset for validation. This process is repeated K times, and each subset is used once as validation data. The results are then averaged to produce a single prediction [41]. The purpose of using cross-validation in machine learning is to accurately predict the performance of machine learning algorithms and evaluate their generalization capabilities. Cross-validation techniques, such as K-fold cross-validation, are essential for evaluating the predictive performance of machine learning models [36,37,38]. It is particularly important because it provides a principled framework for selecting models that maximize generalization performance [20,21]. Cross-validation is used to avoid overfitting and to ensure that model performance is not overly optimistic or pessimistic [41,42,43].

In summary, while using machine learning for modeling, data preprocessing (normalization and augmentation with SMOTE) [44,45,46], modeling, model evaluation (K-fold cross-validation) steps [47,48,49] were systematically applied on Blaine fineness data. All operations performed in the modeling step were performed to ensure model stability and reliability. Python programming language version 3.9, sklearn library version 1.6.1, XGBoost library version 3.0.1 and imbalanced-learn library version 0.13 were used in the modeling phase.

In this study ANN, TabNet, RF, and XGBoost methods were used as modeling methods. Equation (2) was used to determine the coefficients in the Linear Regression (LR) method [50]. In Equation (2), M: Number of samples; p: features; w: coefficients; $y_{i}$: actual value; $\underline{y_{i}}$: predicted value; and x: inputs to adjust the coefficients. In determining the parameters of the modeling methods, the parameters that each method can take were prepared and the most effective parameters of the relevant method on the dataset were selected by trying all parameter combinations on the dataset. The ranking of influential parameters plays a crucial role in both selecting the appropriate GA for the grinding process and enhancing grinding efficiency. Accurate prioritization of these parameters facilitates more environmentally sustainable production under economically favorable conditions, as focusing on the most dominant factors can lead to significant savings in time, energy, and cost. While adjusting the hyperparameters in the ANN method, the number of hidden layers is between 2 and 5, the optimization algorithm is Adam, and SGD and Relu and Linear are used as activation functions. In TabNet method, the learning coefficient was used between 2 × 10^−1^–2 × 10^−3^, step size was between 5 and 20, gamma was between 0.5 and 1.0, and the number of epochs used was 500. In the XGBoost method, when deciding the number of predictors between 3 and 49, squared_error, absolute_error, friedman_mse, and Poisson maximum number of depths was 1–5 for the criterion method used and 0.1–1.0 was for the learning coefficient, hist, exact, and approx metrics for the maximum tree method parameter. In the RF method, when deciding the number of estimators between 3 and 99, the metrics squared_error, absolute_error, friedman_mse, and Poisson maximum depth of 1–5 were used for the criterion method.

(2) $$\begin{array}{c} \sum_{i=1}^{M}{\left(y_{i}-\underline{y_{i}}\right)}^{2}=\sum_{i=1}^{M}{\left(y_{i}-\sum_{j=0}^{p}w_{j}\times x_{ij}\right)}^{2} \end{array}$$

Three different metrics were used to evaluate the results of 3 different modeling methods applied within the scope of this study. Root Mean Square Error (RMSE), Mean Absolute Error (MAE), and Logcosh metrics shown in Equations (3)–(5) were used to evaluate the modeling results.

(3) $$\begin{array}{c} MAE=\frac{1}{n}\;\sum_{i=1}^{n}\left|E_{i-}P_{i\;\;}\right|\; \end{array}$$

(4) $$\begin{array}{c} RMSE={\left[\frac{1}{n}\;\sum_{i=1}^{n}{\left(E_{i-}P_{i\;\;}\right)}^{2}\right]}^{\frac{1}{2}}\; \end{array}$$

(5) $$Logcosh=\sum_{i=1}^{n}\log\left(\cosh\left(\left(E_{i-}P_{i\;}\right)\right)\right)\;\;cosh\left(x\right)=\frac{e^{x}+e^{-x}}{2}$$

Here, E_i_ is the experimental result, P_i_ is the result obtained from our model, and n is the number of data.

**Figure 4:**
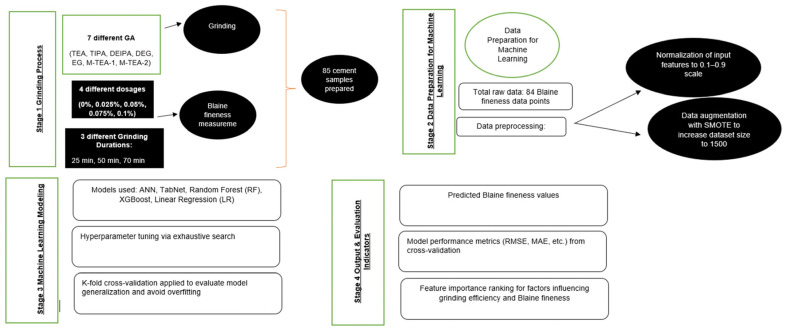
Flowchart of this study.

The flowchart of the study is given in Figure 4.

## 3. Result and Discussion

### 3.1. Assessment of Experimental Results

As illustrated in Figure 1, the Blaine fineness of all cement samples increased with extended grinding time from 25 to 50 min, independent of GA usage. After 25 min of grinding, the control cement (without GA) exhibited the lowest Blaine fineness, whereas the incorporation of GAs led to significant enhancements. Specifically, compared to the control, the Blaine fineness increased by 14%, 9%, 11%, 17%, 19%, 14%, and 16% for TEA, TIPA, DEIPA, DEG, EG, M-TEA-1, and M-TEA-2, respectively. These improvements confirm the efficiency of GA use in short-term grinding, particularly for glycol-based EG, which outperformed others, while TIPA demonstrated the lowest performance in this phase. This trend aligns with findings by Assaad et al. [51], who reported superior short-term grinding efficiency for glycol-based GAs.

Regarding dosage sensitivity, no significant improvement in grinding efficiency was observed with increasing GA dosage beyond optimal levels. For amine-based GAs (TEA, TIPA, DEIPA, M-TEA-1, M-TEA-2), the highest Blaine values were obtained at dosages of 0.025–0.05%, whereas glycol-based GAs (DEG, EG) achieved maximum efficiency at 0.075% and 0.1%, respectively. This suggests that amine-based GAs can reach optimal performance at lower dosages, offering a more dosage-efficient grinding process in the short term.

For the 50 min grinding condition, the control sample again showed the lowest fineness. In contrast, the use of TEA, TIPA, DEIPA, DEG, EG, M-TEA-1, and M-TEA-2 led to fineness increases of 14%, 22%, 10%, 14%, 9%, 14%, and 18%, respectively. Notably, EG, which was the most effective GA at 25 min, became the least efficient at 50 min, whereas TIPA emerged as the most effective. This performance shift is attributed to the ability of TIPA to reduce surface energy through its hydroxyl groups, as supported by research findings [52].

Grinding efficiency was also dosage-dependent in this phase. TIPA exhibited improved performance with increasing dosage, while DEIPA, DEG, and EG reached optimal effectiveness at 0.075%. For TEA, M-TEA-1, and M-TEA-2, an inverse relationship was observed; higher dosages led to reduced grinding efficiency, indicating a threshold beyond which additional GA becomes counterproductive.

At 70 min of grinding, the performance benefit of GAs persisted, with Blaine fineness values ranging from 8% to 25% higher than the control sample. This long-term grinding evaluation confirms that all tested GAs contribute positively to grinding productivity, albeit with varying effectiveness depending on the grinding duration and GA chemistry.

Overall, these findings provide a clearer understanding of the quantitative impact of GA selection on cement fineness. Compared to traditional cement grinding without additives, the inclusion of optimized GA types and dosages can improve Blaine fineness by up to 25%, depending on the grinding duration and chemical structure of the additive. These results offer a measurable and comparative basis for selecting suitable GAs to enhance grinding efficiency in industrial applications.

### 3.2. Evaluation of Modeling Results

Using the results obtained from the experimental data, dominant parameters, determination of the coefficients of the parameters, and modeling studies were carried out using machine and ensemble learning methods of Blaine value, using real and augmented data. Feature importance values for the XGBoost method were used to determine the dominant parameters for grinding efficiency values. The parameters used to determine the dominant parameters with the XGBoost method are presented in Table 3, and the results obtained as a result of the evaluations are presented in Table 4.

The coefficients calculated by the LR method for the grinding efficiency experiment are presented in Table 5. In the table, weights are calculated for seven features (inputs) according to the model created with the augmented data of the grinding efficiency experiment. By using the weights of the features, predictions can be made for data not available in the dataset or available to another researcher. In the grinding efficiency experiment, the weight coefficient of 659.54 or −204.7 for the GA feature was multiplied by the value found in the dataset. After applying this process to the other features, the intercept value in the last column of the table was added to the result. The result obtained was the initial estimate of the data for that experiment.

#### Results for Real Data

The best values obtained from the models on real data for the grinding efficiency experiment are given in Table 6.

**Figure 5:**
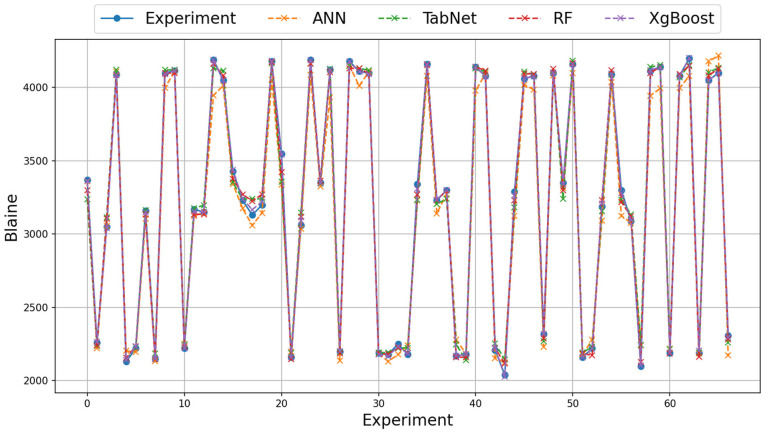
Illustration of experimental and model results on real training data.

**Figure 6:**
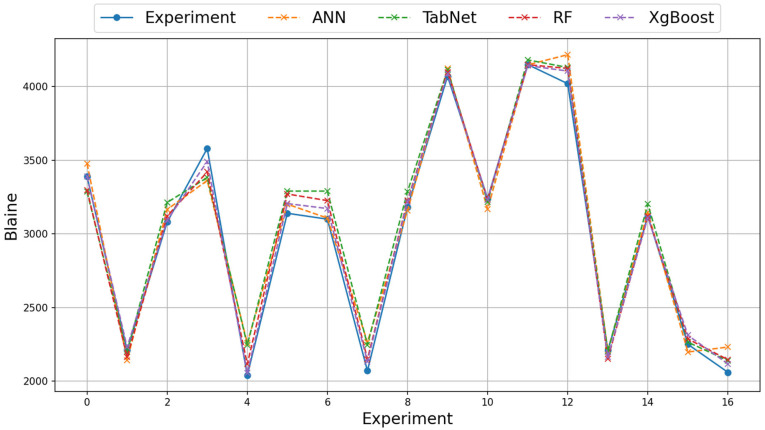
Illustration of experimental and model results on real test data.

For the grinding efficiency experiment, the results obtained on the real and augmented data with the ANN, TabNet, RF, and XGBoost methods are presented in Table 6 and Table 7. The training column shows the results obtained by the method in the learning phase and the test column shows the prediction success of the method in the test phase. The value with the best test result is bolded. As emphasized before, three different metrics, RMSE, MAE, and Logcosh, were used to evaluate the results obtained. As the error metric values approach 0, the prediction success of the method increases, and as they move away, the prediction success decreases. On the real data of the grinding efficiency experiment, the best test results were found to be the XGBoost and RF methods for the RMSE, MAE, and Logcosh metrics, respectively. The TabNet method showed the worst performance in the test results. In terms of metrics, Logcosh achieved the best training and test values in all parameters. This was followed by MAE and RMSE metric results. When the results for three different metrics are examined in all modeling studies, the order of success of the metrics is Logcosh, MAE, and RMSE. In addition to the fact that the modeling results are close to the experimental results, the same ranking of the metric values obtained despite the use of different methods can be presented as evidence of the accuracy of the modeling. The graphical representation of the experimental and model results of the train and test phases with real data is shown in Figure 5 and Figure 6.

**Figure 7:**
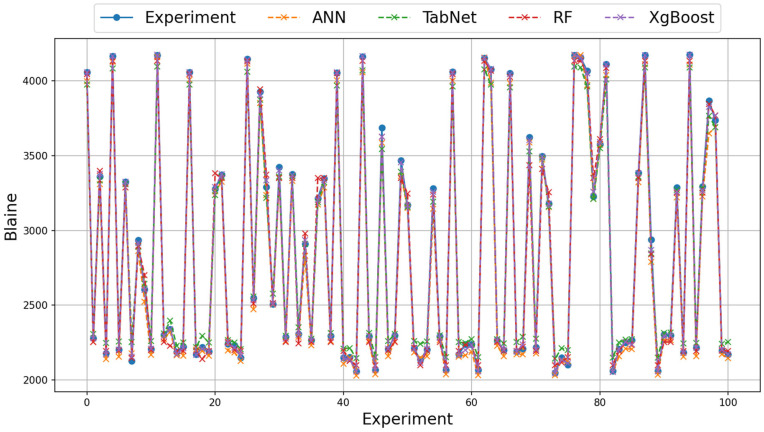
Illustration of experimental and model results on augmented training data (first 100 samples).

**Figure 8:**
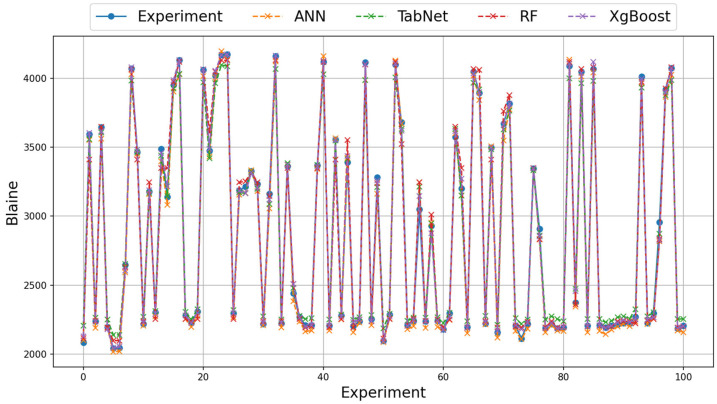
Illustration of experimental and model results on augmented test data (first 100 samples).

On the real data of grinding efficiency experiment, the best test results were found to be the XGBoost, ANN, and RF methods for the RMSE, MAE, Logcosh metrics, respectively. Better results were found in the RMSE metric in the training phase using the ANN method and in the MAE and Logcosh metrics using the RF method. In the test phase, the ANN method was found to be better than the RF method in all metrics. The TabNet method generally showed the worst performance in the test and train results. In terms of metrics, Logcosh achieved the best training and test values for all parameters. This was followed by the MAE and RMSE metric results. When the results for three different metrics are examined across all modeling studies, the order of success of the metrics is Logcosh, MAE, and RMSE. In addition to the fact that the modeling results are close to the experimental results, the same ranking of the metric values obtained despite the use of different methods can be presented as evidence of the accuracy of the modeling. The graphical representation of the experimental and model results of the train and test phases with real data is shown in Figure 7 and Figure 8.

## 4. Conclusions

In this study, Blaine fineness values of cements obtained in the presence of different GA types were modeled using machine learning, transformer-based learning, and ensemble learning methods. The results derived from the obtained data are summarized below, organized under the headings of scientific findings, effective parameters, and application outcomes.

Scientific Findings:

1. The XGBoost method demonstrated superior performance in modeling Blaine fineness values for cements with different grinding aid (GA) types, using both real and augmented datasets. Its efficiency in rapidly generating tree-based models makes it a powerful tool for regression and statistical modeling in cementitious systems.
2. Data augmentation through the Synthetic Minority Over-sampling Technique (SMOTE) significantly improved the predictive accuracy across all modeling methods. Consistency in the ranking of error metrics (Logcosh, MAE, and RMSE) validates the robustness and reliability of the models developed.
3. Ensemble learning methods, such as Random Forest and XGBoost, exhibited strong predictive capability even with limited dataset sizes, whereas Artificial Neural Networks (ANNs) and transformer-based TabNet require larger datasets to achieve comparable success.
4. Parameter importance analysis confirmed grinding time as the most influential factor affecting Blaine fineness, underscoring the physical relevance and accuracy of the modeling approaches.

Effective parameters: milling time, GA dosage, molecular weight, density, pH, number of functional groups, and number of hydrogen bond acceptors.

Applied Results:

1. The integration of advanced machine learning and ensemble methods with data augmentation enables more accurate prediction and optimization of grinding processes in cement production, potentially reducing trial-and-error experimental efforts and accelerating product development cycles.
2. The identification of key parameters influencing grinding efficiency can guide the targeted adjustment of process variables and grinding aid formulations, improving operational efficiency and product quality.

## Figures and Tables

**Table 1:** Some modeling studies on GAs and the grinding process.

Reference	Estimated Parameter	Methodology Used	Highlights
[34]	Modeling and optimization of PEA-combined grinding aids’ effect in the grinding circuit of cement ball mills	Quadratic model for regression analysis	A quadratic regression model was used to investigate the effects of key parameters such as mill utilization rate, Blaine, and GA dosage rate to find the most efficient conditions.
[35]	Grinding roughness prediction model based on an evolutionary artificial neural network.	Genetic algorithm and artificial neural network	Based on analyzing the Back Propagation (BP) network disadvantages of low convergence speed and frequently falling into local minimum value, a roughness prediction model of a BP neural network integrated with a genetic algorithm was proposed.
[36]	Application of statistical and soft computing techniques for the prediction of grinding performance	Artificial neural networks and regression models	In this paper, statistical methods and soft computing techniques, namely regression models completed with analysis of variance and artificial neural networks, respectively, are presented for the estimation of grinding forces and temperature.
[37]	Identification and expert approach to controlling the cement grinding process using artificial neural networks and other non-linear models	NARX models based on feed-forward networks, Elman, Jordan, and Layered Recurrent Network (LRN) recurrent networks, as well as MTL (Multi-Task Learning) and traditional NARX non-linear models	The paper involved conducting preliminary research to explore the identification and control of a multi-dimensional, non-linear, and non-stationary cement grinding process using artificial neural networks and various other non-linear models.
[38]	Empirical mode decomposition-based hybrid ensemble model for electrical energy consumption forecasting of the cement grinding process	Empirical mode decomposition (EMD), moving average filter (MAF), least squares support vector regression (LSSVR), and quadratic exponential smoothing (QES)	Forecasting the electrical energy consumption of the cement grinding process remains a difficult task due to the intrinsic complexity and irregularity of its time series. To solve this difficulty and improve the prediction accuracy, a novel hybrid model was proposed based on the “decomposition-prediction-integration” methodology.

**Table 2:** Some chemical properties of the GAs used.

Types of GAs	Molecular Weight (g/mol)	Density (g/cm^3^)	pH. 25 °C	Hydrogen Bonds	Number of Functional Groups
TEA	148.19	1.095	10.5	4	3
TIPA	191.27	1.124	10.8	4	3
DEIPA	163.20	1.079	9.7	3	3
DEG	106.12	1.118	7.2	3	2
EG	62.07	1.26	8.2	3	2
M-TEA1	207.00	1.206	5.6	4	3
M-TEA2	327.00	1.166	2.63	4	3

**Table 3:** Parameters used in experiments for the RF method.

Experiment Data	Parameters			
	Estimator	Depth	Learning Rate	Tree Method
Real	47	4	0.3	hist
Augmented	47	4	0.3	exact

**Table 4:** Parameters affecting grinding efficiency values (feature importance values).

Model	GA Dosage (%)	Molecular Weight	Density	pH	Number of Hydrogen Bond Acceptors	Number of Functional Groups	Grinding Time (s)
Real Data	0.000749	0.005852	0.004484	0.002404	0.000531	0.000001	0.98598
Sorting	5	2	3	4	6	7	1
Augmented Data	0.001604	0.004744	0.003322	0.002009	0.001754	0.000001	0.986568
Sorting	6	2	3	4	5	7	1

**Table 5:** Equationization of the calculated coefficients.

Experiment	Coefficients							
	GA	Molecular Weight	Density	pH	HBA	FGA	GT	Intercept
Blaine(Real Data)	−204.7	−0.53	−1764.46	−36.75	83.67	83.67	40.37	3049.43
Blaine(Augmented Data)	659.54	−0.1	−1192.59	−25.63	24.29	24.29	41.23	2561.88

**Table 6:** Best values obtained from models on real data for the grinding efficiency experiment.

Blaine	MAE		RMSE		LogCosh	
Model	Train	Test	Train	Test	Train	Test
ANN	71.7741	90.4075	91.0063	117.8307	89.7182	71.0810
TabNet	40.6502	98.1536	56.0825	119.0298	39.9612	97.4604
RF	29.1276	65.7865	38.5301	79.8695	28.4370	46.7729
XGBoost	7.1568	39.4859	10.9029	49.0680	22.1620	22.6828

**Table 7:** The best values obtained from the models on the augmented data for the grinding efficiency experiment.

Blaine	MAE		RMSE		LogCosh	
Model	Train	Test	Train	Test	Train	Test
ANN	43.3705	39.9442	52.6161	49.9826	42.6816	39.2522
TabNet	53.9081	52.8663	62.6867	61.9604	53.2175	52.1758
RF	38.1095	42.5909	56.5102	62.9739	39.8402	40.0979
XGBoost	11.2431	21.0384	16.6611	33.7379	10.5850	15.4846

## Data Availability

The original contributions presented in this study are included in the article. Further inquiries can be directed to the corresponding author.

## References

[1] Kobya V., Karakuzu K., Mardani A., Felekoğlu B., Ramyar K. (2023). Combined interaction of PCE chains lengths, C3A and water content in cementitious systems. Constr. Build. Mater..

[2] Şahin H.G., Biricik Ö., Mardani-Aghabaglou A. (2022). Polycarboxylate-based water reducing admixture–clay compatibility; literature review. J. Polym. Res..

[3] Lei L., Hirata T., Plank J. (2022). 40 years of PCE superplasticizers-History, current state-of-the-art and an outlook. Cem. Concr. Res..

[4] Ma Y., Sha S., Zhou B., Lei F., Liu Y., Xiao Y., Shi C. (2022). Adsorption and dispersion capability of polycarboxylate-based superplasticizers: A review. J. Sustain. Cem.-Based Mater..

[5] Özen S., Altun M.G., Mardani-Aghabaglou A., Ramyar K. (2021). Effect of main and side chain length change of polycarboxylate-ether-based water-reducing admixtures on the fresh state and mechanical properties of cementitious systems. Struct. Concr..

[6] Sha S., Wang M., Shi C., Xiao Y. (2020). Influence of the structures of polycarboxylate superplasticizer on its performance in cement-based materials-A review. Constr. Build. Mater..

[7] Yiğit B., Salihoğlu G., Mardani-Aghabaglou A., Salihoğlu N.K., Özen S. (2020). Recycling of sewage sludge incineration ashes as construction material. J. Fac. Eng. Archit. Gazi Univ..

[8] Aytekin B., Mardani-Aghabaglou A. (2022). Sustainable materials: A review of recycled concrete aggregate utilization as pavement material. Transp. Res. Rec..

[9] Giergiczny Z. (2019). Fly ash and slag. Cem. Concr. Res..

[10] Mathapati M., Amate K., Prasad C.D., Jayavardhana M.L., Raju T.H. (2022). A review on fly ash utilization. Mater. Today Proc..

[11] Becerra-Duitama J.A., Rojas-Avellaneda D. (2022). Pozzolans: A review. Eng. Appl. Sci. Res..

[12] Chipakwe V., Semsari P., Karlkvist T., Rosenkranz J., Chelgani S.C. (2020). A critical review on the mechanisms of chemical additives used in grinding and their effects on the downstream processes. J. Mater. Res. Technol..

[13] Hao S.H., Liu B.H., Yan X.Y. (2017). Review on research of cement grinding aids and certain problems. Key Eng. Mater..

[14] Nthiga Njiru E., Wachira Muthengia J., Mulwa Munyao O., Karanja Mutitu D., Munyao Musyoki D. (2023). Review of the Effect of Grinding Aids and Admixtures on the Performance of Cements. Adv. Civ. Eng..

[15] Assaad J.J., Asseily S.E., Harb J. (2010). Use of cement grinding aids to optimise clinker factor. Adv. Cem. Res..

[16] Prziwara P., Breitung-Faes S., Kwade A. (2018). Impact of grinding aids on dry grinding performance, bulk properties and surface energy. Adv. Powder Technol..

[17] Assaad J.J., Issa C.A. (2014). Effect of clinker grinding aids on flow of cement-based materials. Cem. Concr. Res..

[18] Kaya Y., Kobya V., Mardani A., Assaad J.J. (2024). Effect of Modified Triethanolamine on Grinding Efficiency and Performance of Cementitious Materials. Talanta Open.

[19] Durgun M.Y., Özen S., Karakuzu K., Kobya V., Bayqra S.H., Mardani-Aghabaglou A. (2022). Effect of high temperature on polypropylene fiber-reinforced mortars containing colemanite wastes. Constr. Build. Mater..

[20] Jolicoeur J., Morasse S., Sharman J., Tagnit-Hamou A., Slim F., Page M. Polyol-type compounds as clinker grinding aids: Influence of powder fluidity and on cement hydration. Proceedings of the 12th International Congress on the Chemistry of Cement.

[21] Zhang T., Gao J., Hu J. (2015). Preparation of polymer-based cement grinding aid and their performance on grindability. Constr. Build. Mater..

[22] Sun Z., Liu H., Ji Y., Pang M. (2020). Influence of glycerin grinding aid on the compatibility between cement and polycarboxylate superplasticizer and its mechanism. Constr. Build. Mater..

[23] Kobya V., Kaya Y., Mardani-Aghabaglou A. (2022). Effect of amine and glycol-based grinding aids utilization rate on grinding efficiency and rheological properties of cementitious systems. J. Build. Eng..

[24] Zhao J., Wang D., Wang X., Liao S. (2015). Characteristics and mechanism of modified triethanolamine as cement grinding aids. J. Wuhan Univ. Technol.-Mater. Sci. Ed..

[25] Kaya Y., Kobya V., Mardani A. (2024). Evaluation of fresh state, rheological properties, and compressive strength performance of cementitious system with grinding aids. J. Appl. Polym. Sci..

[26] Sutton R.S., Barto A. (1998). Reinforcement learning: An introduction. IEEE Trans. Neural Netw..

[27] Mardani-Aghabaglou A., Özen S., Altun M.G. (2018). Durability performance and dimensional stability of polypropylene fiber reinforced concrete. J. Green Build..

[28] Ai Y., Ye C., Liu J., Zhou M. (2025). Study on the evolution processes of keyhole and melt pool in different laser welding methods for dissimilar materials based on a novel numerical model. Int. Commun. Heat Mass Transf..

[29] Khan I.U., Ouaissa M., Ouaissa M., Fayaz M., Ullah R. (2024). Artificial Intelligence for Intelligent Systems: Fundamentals, Challenges, and Applications.

[30] Zhao Z., Fan W., Li J., Liu Y., Mei X., Wang Y., Wen Z., Wang F., Zhao X., Tang J. (2024). Recommender systems in the era of large language models (llms). IEEE Trans. Knowl. Data Eng..

[31] Bouguettaya A., Zarzour H., Kechida A., Taberkit A.M. (2022). Machine learning and deep learning as new tools for business analytics. Handbook of Research on Foundations and Applications of Intelligent Business Analytics.

[32] Hosni M., Abnane I., Idri A., de Gea J.M.C., Alemán J.L.F. (2019). Reviewing ensemble classification methods in breast cancer. Comput. Methods Programs Biomed..

[33] Yüksel C., Mardani-Aghabaglou A., Beglarigale A., Yazıcı H., Ramyar K., Andiç-Çakır Ö. (2016). Influence of water/powder ratio and powder type on alkali–silica reactivity and transport properties of self-consolidating concrete. Mater. Struct..

[34] Fatahi R., Pournazari A., Ghorbani H., Shah M.P., tazik S.H., Abdollahi H. (2023). Modeling and optimization of pea- combined grinding aids effect in grinding circuit of cement ball mill. Res. Sq..

[35] Chen L.Q., Guo J.L., Yang X., Chi J., Zhao X. (2013). Grinding roughness prediction model based on evolutionary artificial neural network. Comput. Integr. Manuf. Syst..

[36] Karkalos N.E., Markopoulos A.P., Dossis M.F. (2015). Application of statistical and soft computing techniques for the prediction of grinding performance. J. Robot Mech. Eng. Resr..

[37] Pawuś D., Paszkiel S. (2024). Identification and Expert Approach to Controlling the Cement Grinding Process Using Artificial Neural Networks and Other Non-Linear Models. IEEE Access.

[38] Liu Z., Wang X., Zhang Q., Huang C. (2019). Empirical mode decomposition based hybrid ensemble model for electrical energy consumption forecasting of the cement grinding process. Measurement.

[39] Chawla N.V., Bowyer K.W., Hall L.O., Kegelmeyer W.P. (2002). SMOTE: Synthetic minority over-sampling technique. J. Artif. Intell. Res..

[40] (2000). Cement: Composition, Specifications and Conformity Criteria, Part 1: Common Cements.

[41] Dake D.K., Gyimah E. (2022). Using sentiment analysis to evaluate qualitative students’ responses. Educ. Inf. Technol..

[42] Ramezan C.A., Warner T.A., Maxwell A.E. (2019). Evaluation of sampling and cross-validation tuning strategies for regional-scale machine learning classification. Remote Sens..

[43] Krstajic D., Buturović L., Leahy D.E., Thomas S. (2014). Cross-validation pitfalls when selecting and assessing regression and classification models. J. Cheminformatics.

[44] Poudyal R., Paneru B., Paneru B., Giri T., Paneru B., Reynolds T., Poudyal K.N., Dangi M.B. (2025). Exploring Cement Production’s Role in GDP Using Explainable AI and Sustainability Analysis in Nepal. Case Stud. Chem. Environ. Eng..

[45] Zhang A., Yu H., Huan Z., Yang X., Zheng S., Gao S. (2022). SMOTE-RkNN: A hybrid re-sampling method based on SMOTE and reverse k-nearest neighbors. Inf. Sci..

[46] Barratt S., Sharma R. (2018). Optimizing for generalization in machine learning with cross-validation gradients. arXiv.

[47] Granholm V., Noble W.S., Käll L. (2012). A cross-validation scheme for machine learning algorithms in shotgun proteomics. BMC Bioinformatics.

[48] Mardani-Aghabaglou A., Öztürk H.T., Kankal M., Ramyar K. (2021). Assessment and prediction of cement paste flow behavior; Marsh-funnel flow time and mini-slump values. Constr. Build. Mater..

[49] Mardani-Aghabaglou A., Kankal M., Nacar S., Felekoğlu B., Ramyar K. (2021). Assessment of cement characteristics affecting rheological properties of cement pastes. Neural Comput. Appl..

[50] Montgomery D.C., Peck E.A., Vining G.G. (2012). Introduction to Linear Regression Analysis.

[51] Assaad J.J., Asseily S.E., Harb J. (2009). Effect of specific energy consumption on fineness of portland cement incorporating amine or glycol-based grinding aids. Mater. Struct..

[52] Mishra R.K., Weibel M., Müller T., Heinz H., Flatt R.J. (2017). Energy-effective grinding of inorganic solids using organic additives. Chim. Int. J. Chem..

